# A Parsimony Approach to Biological Pathway Reconstruction/Inference for Genomes and Metagenomes

**DOI:** 10.1371/journal.pcbi.1000465

**Published:** 2009-08-14

**Authors:** Yuzhen Ye, Thomas G. Doak

**Affiliations:** 1 School of Informatics, Indiana University, Bloomington, Indiana, United States of America; 2 Biology Department, Indiana University, Bloomington, Indiana, United States of America; King's College London, United Kingdom

## Abstract

A common biological pathway reconstruction approach—as implemented by many automatic biological pathway services (such as the KAAS and RAST servers) and the functional annotation of metagenomic sequences—starts with the identification of protein functions or families (e.g., KO families for the KEGG database and the FIG families for the SEED database) in the query sequences, followed by a direct mapping of the identified protein families onto pathways. Given a predicted patchwork of individual biochemical steps, some metric must be applied in deciding what pathways actually exist in the genome or metagenome represented by the sequences. Commonly, and straightforwardly, a complete biological pathway can be identified in a dataset if at least one of the steps associated with the pathway is found. We report, however, that this naïve mapping approach leads to an inflated estimate of biological pathways, and thus overestimates the functional diversity of the sample from which the DNA sequences are derived. We developed a parsimony approach, called MinPath (*Min*imal set of *Path*ways), for biological pathway reconstructions using protein family predictions, which yields a more conservative, yet more faithful, estimation of the biological pathways for a query dataset. MinPath identified far fewer pathways for the genomes collected in the KEGG database—as compared to the naïve mapping approach—eliminating some obviously spurious pathway annotations. Results from applying MinPath to several metagenomes indicate that the common methods used for metagenome annotation may significantly overestimate the biological pathways encoded by microbial communities.

## Author Summary

Even though there is only a single large biological network within any cell and all pathways are to some extent connected, the partition of the entire cellular network into smaller units (e.g., KEGG pathways) is extremely important for understanding biological processes. Biological pathway reconstruction, therefore, is essential for understanding the biological functions that a newly sequenced genome encodes and recently for studying the functionality of a natural environment via metagenomics. The common practice of pathway reconstruction in metagenomics first identifies functions encoded by the metagenomic sequences and then reconstructs pathways from the annotated functions by mapping the functions to reference pathways. To address the issues of both incomplete data (e.g., metagenomes, unlike individual genomes, are most likely incomplete) and pathway redundancy (e.g., the same function is involved in multiple pathway units), we formulate a parsimony version of the pathway reconstruction/inference problem, called MinPath (*Min*imal set of *Path*ways): given a set of reference pathways and a set of functions that can be mapped to one or more pathways, MinPath aims at finding a minimum number of pathways that can explain all functions. MinPath achieves a more conservative, yet more faithful, estimation of the biological pathways encoded by genomes and metagenomes.

## Introduction

Microbial whole genome sequencing has become a routine practice in recent years, because of the rapid advances of DNA sequencing technologies [1]. One of the first analyses that biologists attempt, once they obtain a complete genome sequence, is to *reconstruct* the biological pathways encoded by the organism, which is usually accomplished *in silico* by mapping the protein coding genes onto reference pathway collections, such as KEGG [2] or SEED [3], based on their homology to reference genes with previously characterized functions. For example, KAAS, the pathway annotation system based on the KEGG database [4], first annotates K numbers (each K number represents an ortholog group of genes, and is directly linked to an object (a biochemical step) in the KEGG pathway map), and then reconstructs pathways based on the assigned K numbers. Similarly, the RAST server (and MG-RAST) first annotates FIG families and then maps the identified FIG families onto the SEED subsystems [5],[6]. These automatic methods are promising for the analysis of most genomes, although they may leave “holes” in the reconstructed pathways, due to either *missing* genes (i.e. the genes are non-homologous to reference genes of the same specific functions, and thus cannot be identified by a homology-based method, or were simply not annotated as ORFs by annotation pipelines) [7], or alternative and novel pathways (i.e. the target organism adopts variant pathways, which are different from the reference pathway, to accommodate a specific niche or lifestyle) [8]. After all, many bacterial genomes have fewer than 60% of their genes assigned to a proposed function [9],[10].

We note that *pathway reconstruction* is essential for understanding the biological functions that a newly sequenced genome encodes. For instance, in a recently published report, the coupling of N_2_ fixation to cellulolysis was revealed within protist cells in the termite gut, based solely on the *in silico* pathway reconstruction of the complete genome sequence of a bacterial endosymbiont [11].

Moreover, pathway reconstruction based on some new high throughput techniques must provide conclusions from explicitly incomplete information, which poses fresh challenges. For example, in a typical *proteomics* experiment, the proteins represent a particular biological sample collected under a specific physiological condition or from a specific tissue (e.g. from yeast cells after the heat shock), which are in high enough abundance to be identified by tandem mass spectrometry [12],[13]. Based on these data, one may ask, what biological pathways were activated (or suppressed) under the physiological condition? A similar, but more complicated case is pathway analysis of *metagenomic* data, to characterize the aggregate metabolic processes of microbial communities in a given environment [14]. Metagenomic profiling data can be viewed as a sampling of the genomic sequences from many kinds of microbes living in a specific environment. Again, the incompleteness of the data makes it difficult to reconstruct the entire pathways encoded by a metagenome. Nevertheless, it is becoming routine to “reconstruct” pathways for proteomic [15] and metagenomic data [16],[17], by best similarity matches (often derived from BLAST searches): a pathway is inferred to be absent or present in a dataset if highly confident homolog protein hits identify one or more of the protein functions associated with the pathway in other organisms.

In addition to the problems that arise from incomplete data, existing methods of pathway reconstruction or inference may over-estimate the number of pathways because of redundancy in the protein-pathway, at four levels. First, different pathways may share the same biological functions. The partition of pathways (as the entire cellular network is partitioned into several hundreds of biological pathway entities in KEGG database) is extremely important for understanding of biological processes, even though there is only a single large biological network within any cell and all pathways are to some extent connected [18]. It is not surprising that many pathways defined in the pathway databases are overlapping. Second, some proteins carry out multiple biological functions [19], *e.g.* through different protein domains, active sites, or substrate specificities. Third, neither organisms nor communities are closed boxes, and the products or intermediates of pathways may be exogenously supplied. Finally, homology-based protein searching may map one protein to multiple homologous proteins with different biological functions (i.e. paralogous proteins). In summary, it cannot be safely concluded that a pathway is present, even if one or more proteins are mapped to it.

Even for single complete genomes, pathway reconstruction does not always give a clear picture of the biological functions in an organism, and human curation and experimental verification is often needed [20],[21]. We illustrate this by a rather extreme example found in the pathway analysis of the human genome. The KEGG pathway annotation of the human genome includes the reductive carboxylate cycle, with proteins annotated to 6 steps in this pathway (http://www.genome.jp/kegg-bin/show_organism?menu_type=pathway_maps&org=hsa) (as of July 2^nd^, 2009). The Calvin cycle is the most common method of carbon fixation, while the reductive carboxylate cycle is an alternative carbon fixation pathway, currently found only in certain autotrophic microorganisms. In fact, the reductive carboxylate cycle is essentially the reverse of the Krebs cycle (citric acid or tricarboxylic acid cycle), the final common pathway in aerobic metabolism for the oxidation of carbohydrates, fatty acids and amino acids, so they share reactions and functional roles. For this reason, the proteins responsible for the normal function of the Krebs cycle can be mistakenly taken as evidence for the existence of a reductive carboxylate cycle in the human genome.

**Figure 1:**
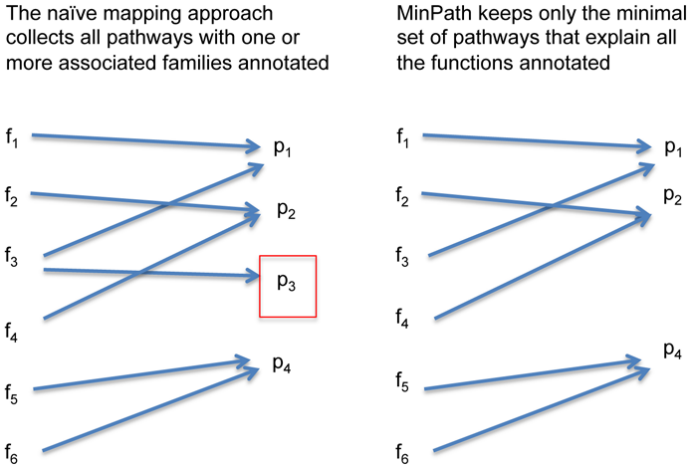
Schematic illustration of the MinPath method. Assume 6 families (or orthologous groups, f_1_, …, f_6_) are identified from a given sample of genes (e.g., the genes could be from a genome, or sampled from a metagenome). The naïve mapping approach (shown on the left) will lead to a reconstruction with 4 pathways annotated (p_1_, p_2_, p_3_, and p_4_). Due to the overlapping nature of the biological pathways (see text for more details), pathway p_3_ shares function f_3_ with pathway p_2_. We claim that only three pathways, p_1_, p_2_, and p_3_ are sufficient to explain the existence of the 6 families annotated in the dataset, and a conservative reconstruction of pathways should have only 3 pathways (shown on the right). As we show in the paper, such a conservative estimation of pathways provides a more reliable estimation of the functional diversity of a sample.

Here we propose a pathway reconstruction/inference method in which we do not attempt to reconstruct entire pathways from a given set of protein sequences (e.g. identified in a proteomics experiment, or encoded by the sequences sampled in a metagenomic project), but to determine the minimal set of biological pathways that must exist in the biological system to explain the input protein sequences sampled from it. In this context, we note *pathway inference* might be a more suitable terminology than *pathway reconstruction*. However, considering that pathway inference has been used in a different context to infer networks or pathways from gene express data [22], and pathway reconstruction is commonly used in the field, we use both pathway inference and pathway reconstruction in this paper. To address the issues of both incomplete data, and pathway redundancy, we formulate a parsimony version of the pathway reconstruction/inference problem, called MinPath (*Min*imal set of *Path*ways), which can be roughly described as the following: given a set of reference pathways and a set of proteins (and their predicted functions) that can be mapped to one or more pathways, we attempt to find the minimum number of pathways that can explain all proteins (functions) (see Fig. 1). Although this problem is NP-hard in general, we provide an integer programming (IP) framework to solve it.

We focus on analyzing complete genomes in this study because there is a relatively good understanding of the pathways that actually exist in organisms with completely sequenced genomes (as compared to the emerging metagenomes), making this analysis a good test of our method. Besides, the pathway annotations of these genomes are still far from perfection, as in the example of a carbon fixation pathway in the human genome (as well as chickens, mosquitoes, etc). We also applied MinPath to the analyses of several metagenomic datasets, to demonstrate the potential applications of MinPath in metagenome annotation.

## Results

We first revisited the pathway reconstruction of individual genomes using MinPath. The results show that MinPath gave a conservative, but reliable estimation of the pathways of a genome, and therefore the functional ability/diversity encoded by a genome. In addition, MinPath found suspicious pathways in the KEGG database. We then applied MinPath to a set of metagenomic datasets, and the results indicate that the current estimation of functional diversity/ability of studied microbial communities might be overestimated.

### Pathway Reconstruction for Genomes

#### Overview of the performance of MinPath

**Figure 2:**
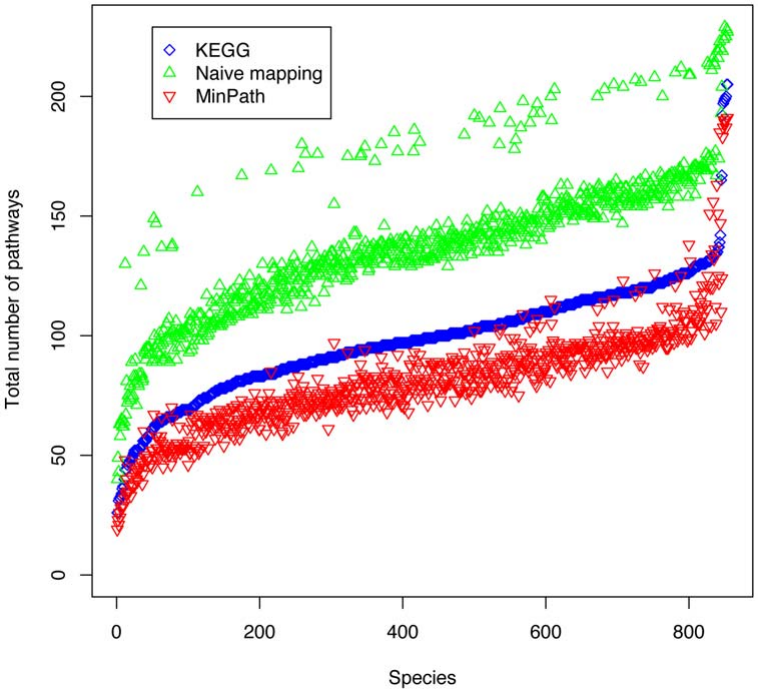
Comparison of the number of pathways reconstructed for various genomes by different methods. The coloring schema is as following: MinPath (red triangles), naïve mapping approach (green), and the pathway annotation maintained in KEGG database after human evaluation (blue).

The function annotations of the genes from individual genomes were extracted from the KEGG database, and used as input for MinPath to reconstruct the pathways encoded by each genome. A total of 854 genomes were studied, and the overall performance of MinPath is shown in Fig. 2, compared with the curated KEGG pathways and the pathway reconstructions produced by the naïve mapping approach (see METHODS for details). The comparison shows that MinPath gives an estimation of functional diversity (measured by the number of pathways constructed) that is closer to the curated KEGG database, as compared to simple pathway construction based on the appearance of families. MinPath gives a more conservative estimation of the pathways than even KEGG in most genomes (with fewer annotated biological pathways), but we would like to argue that even some of the pathways collected in KEGG should be removed (such as the ascorbate and aldarate metabolism pathway in human, as we discuss below).

#### The human genome

For the human genome, there are 205 predicted KEGG pathways (as of December 2008), while the naïve mapping approach identifies 227 pathways. MinPath identified only 191 pathways—these pathways are necessary and sufficient to explain all the annotated human proteins in the KEGG database.

Many of the pathways that are identified by the naïve mapping approach are spurious and are not curated in the KEGG database (e.g. the penicillin and cephalosporin biosynthesis pathway, and the two-component general and the type II secretion systems), indicating that MinPath can be applied to remove pathways that are otherwise mistakenly annotated using the naïve mapping approach. More examples are listed in the supplementary website.

**Figure 3:**
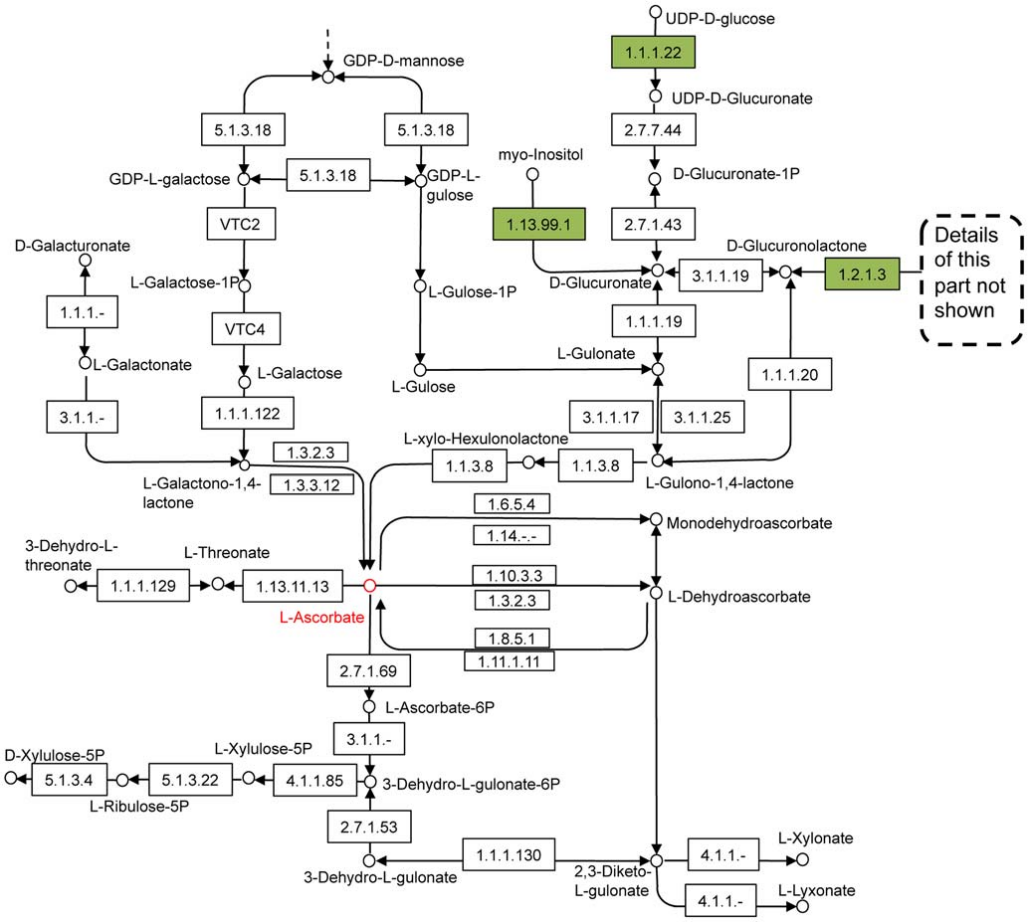
The ascorbate and aldarate metabolism pathway, eliminated by MinPath. The diagram was prepared based on the corresponding KEGG pathway (ID = 00053), and only part of the pathway is shown for clarity. The three enzymes that are annotated in the human genome are highlighted in green, even though none of these enzymes are unique to this pathway.

Some of the pathways that are curated in the KEGG database are marked by MinPath as spurious (see Table 1). For example, the ascorbate and aldarate metabolism pathway (Fig. 3) is annotated in KEGG as a biological pathway in human, but not by MinPath. In humans there are only three functions (out of 24) annotated for this pathway and these three functions are not unique to the pathway: EC 1.2.1.3 (aldehyde dehydrogenase 2 family) is involved in 15 other pathways, EC 1.1.1.22 (UDP-glucose dehydrogenase) is involved in three other pathways, and myo-inositol oxygenase (EC:1.13.99.1) is involved in both this pathway and the inositol phosphate metabolism pathway. Based on the sparseness of the genes assigned to this pathway and their ubiquitous nature, and the fact that humans require vitamin C in the diet, we believe that the ascorbate and aldarate metabolism pathway should be removed from the pathways reconstructed for the human genome.

**Table 1:** Selected spurious pathways of the human genome that are incorrectly identified by the naïve mapping approach.

KEGG ID	Pathway description	Possible reason for being falsely identified by the naïve mapping approach	Removed by MinPath?	Additional notes
00053	ascorbate and aldarate metabolism	pathway redundancy (same function involves in multiple pathways)	yes	humans can not synthesize ascorbic acid (vitamin C)
00290	valine, leucine and isoleucine biosynthesis	pathway redundancy	yes	all three are essential amino acids in humans
00521	streptomycin biosynthesis^a^	pathway redundancy	yes	see table note^a^
00720	reductive carboxylate cycle	pathway redundancy	yes	it is a CO_2_ fixation pathway found in photosynthetic bacteria

a Steptomycin biosynthesis is not listed for the human genome (http://www.genome.jp/kegg-bin/show_organism?menu_type=pathway_maps&org=hsa) in KEGG; but there are 5 functional roles from this pathway annotated in the human genome based on the KEGG annotation, including K00844, K01092, K01710, K01835, and K01858.

#### The Escherichia coli genome

MinPath identified 98 biological pathways that are sufficient to explain all the identified functions encoded by the *E.coli* genome. It is a conservative estimation, as compared to the 125 pathways for *Escherichia coli K-12 MG1655* collected in the KEGG database, and 158 pathways that have at least one or more associated functions identified in the genome. Refer to the supplementary webpage for the details of the pathway reconstructions and their comparison for *E.coli*. It is obvious that the naïve mapping approach leads to an inflated estimate of biological pathways in *E.coli*—the list even includes several biological pathways involved with human cancer, including renal cell carcinoma (pathway ID 05211), prostate cancer (pathway ID 05215), and bladder cancer (pathway ID 05219). These pathways were wrongly annotated because one or more predicted functions in these pathways are also involved in other pathways. For example, fumarate hydratase (ko:K01679) is involved in the renal cell carcinoma pathway, as well as in the citrate cycle, a fundamental pathway present in most bacteria. Based on the identification of this enzyme alone, the naïve mapping approach predicted the presence of the renal cell carcinoma pathway in *E. coli*, which obviously cannot be true. MinPath removed these spurious pathways from the list of constructed pathways, without human curation.

We argue that KEGG predictions also overestimate the biological pathway encoded by *E. coli* genome, e.g. the mitochondrial fatty acid elongation pathway and the bile acid biosynthesis pathway (see Table 2).

**Table 2:** Selected spurious pathways of the *E. coli* genome (collected in KEGG) eliminated by MinPath.

KEGG ID	Pathway description	Functions involved	Removed by MinPath?	Justification	Additional notes
00062	fatty acid elongation in mitochondria	K00022	yes	K00022 is shared by this pathway and 6 other pathways	*E.coli* has no mitochondria
00521	bile acid biosynthesis	K00001 K00632	yes	K00001 is shared by several other pathways, including the glycolysis pathway; K00632 is shared by the fatty acid metabolism pathway and others.	bile acids are steroid acids found predominantly in the bile of mammals

### Pathway Reconstruction for Metagenomes

We used MinPath to re-analyze the biological pathways of several metagenomes [17], which were previously analyzed by a naïve mapping approach. The results are summarized in Table 3. We used both the KEGG and SEED databases in this experiment. For KEGG pathways, we did local BLAST searches, using the criteria as shown in [16] for KO family identification. For SEED subsystems, the FIG annotations were downloaded from the MG-RAST server (http://metagenomics.theseed.org/).

**Table 3:** Comparison of biological pathway reconstruction based on MinPath and the naïve mapping approach for selected metagenomes^a^.

Environmental samples	Naïve mapping (KEGG)^c^	MinPath (KEGG)	Naïve mapping (SEED)^d^	MinPath (SEED)
Coral-Mic (7)^b^	188/232^e^	109/171	497/629	186/392
Coral-Vir (6)	174/211	105/140	594/667	285/441
Marine-Mic (8)	221/236	146/174	695/730	488/577
Marine-Vir (10)	213/236	154/175	680/733	507/599
Freshwater-Mic (4)	196/220	137/165	678/739	460/601
Freshwater-Vir (4)	113/154	57/90	392/559	112/283
Hyper-saline-Mic (9)	196/221	146/170	724/763	558/650
Hyper-saline-Vir (12)	164/181	105/137	613/697	347/510

a metagenomes sampled from different environments [17] (-Mic, and -Vir are for microbial and viral metagenomes, respectively, as shown in the table).

b microbial metagenomes sampled from coral, with the total number of sequencing datasets shown in the brackets.

c based on the KEGG pathways (the KEGG database used in this study was downloaded in Dec, 2008, which has 345 pathways).

d based on the SEED subsystems (we used FIGfams release 6, which has more subsystems than reported in [17], and the total number of subsystems included is 898).

e the two numbers present the total number of pathways (or subsystems) found in at least two of the datasets (e.g., two out of 7 for Coral-Mic), and in at least one of the datasets for each environmental location, respectively.

For all the datasets we tested, MinPath reduced the total number of annotated pathways (or subsystems) significantly (as shown in Table 3). For example, for the metagenome sampled from a coral microbial community (Coral-Mic), there are in total 232 KEGG biological pathways annotated in at least one of the 7 sequencing datasets. Based on MinPath, however, only 160 KEGG biological pathways are sufficient to explain all the functions predicted for these datasets. These results indicate that the naïve mapping of the biological pathways from predicted functions may overestimate the biological pathways (so the functional diversity) of those microbial communities, and we need to be cautious when interpreting the results from such an analysis [16],[17].

We also show the details of pathway reconstruction for a single sequence dataset from the coral biome (4440319.3.dna.fa). The naïve mapping approach identified 224 KEGG pathways, whereas MinPath identified only 143 KEGG pathways. The pathways eliminated by MinPath include the inositol metabolism pathway, the androgen and estrogen metabolism pathway, the caffeine metabolism pathway, etc (see more examples at the supplementary website). Obviously, comparisons of microbial communities or other biomes will be more telling if spurious pathways are eliminated, and our results suggest that as many as 40% of the 224 pathways could be wrong.

## Discussion

We have developed the MinPath approach to provide more conservative—but more reliable—estimations of biological pathways from a sequence dataset, and applied this approach to revisit the biological pathway reconstruction problem for genomes as well as metagenomes. Our results show that without further post-processing of the reconstructed pathways, the naïve mapping strategy may overestimate the biological pathways that are encoded by a genome or metagenome, which could jeopardize any conclusions drawn from the constructed biological pathways (such as the metabolic diversity/capacity of an environmental microbial or viral community, as measured by the Shannon Index) [16],[17], or other downstream analysis based on constructed pathways [23]. It was noted in [16] that most of the microbial communities in that study were approaching saturation for known pathways: more conservative estimates of pathways for each environment may allow real functional differences between the samples to be detected.

Note that MinPath is not designed to directly improve the still imperfect definition of pathways and/or functions in databases such as KEGG or SEED. For example, as a result of how some pathways are grouped in the KEGG database, peptidoglycan biosynthesis is listed for the human genome by KEGG annotation and MinPath does not eliminate this pathway from the list of annotated pathways from human genome. In this sense, efforts are still needed to improve the elucidation and annotation of extent biochemical pathways. But given a database of reference pathways, we feel that MinPath provides a sensible method for inferring the pathways represented in biological sequence samples.

## Materials and Methods

First we will briefly describe the naïve mapping approach that is commonly used in current automatic biological pathway reconstruction services (e.g., the KAAS and RAST servers), as well as for pathway reconstruction for metagenomic sequences. Then we present a novel minimal pathway reconstruction approach based on a simple yet efficient algorithm for solving this problem.

### The Naïve Mapping Approach to Pathway Reconstruction

Pathway reconstruction has become routine in functional annotation of genomes and metagenomes, in which KEGG pathways (or other biological pathways such as SEED subsystems) are reconstructed based on homology. KEGG and SEED databases collect pathways (or subsystems) curated by experts, each pathway/subsystem consisting of a series of functional roles (enzymes, transporters, etc). Pathway reconstruction consists of two key steps: (1) predicting the functions (represented by protein families) of proteins encoded by the DNA sequences, which is often achieved by similarity searching of the predicted proteins against reference proteins from previously characterized genomes; and (2) predicting the presence or absence of pathways in the query dataset, based on the identified functions associated to the pathways. Conventional pathway reconstruction usually adopts simple criterion in this second step (herein referred to as the naïve mapping approach), i.e., a pathway is considered to be present if one or more functions in the pathway are identified in the first step. We have shown in this paper that this approach may lead to the identification of spurious pathways and an overestimation of functional ability, which motivated us to develop a novel approach to pathway reconstruction based on the parsimony principle presented below.

### Minimal Pathway Reconstruction Problem

We define the *minimal pathway reconstruction problem* as the following: given a list of functions annotated for a set of genes (which can be an incomplete set, as we encounter in metagenomic analysis, or a nearly complete set, as in complete genome analysis), find the *minimal* set of pathways that include all given functions (see Fig 1). Note that this formulation is different from the conventional formulation of the pathway reconstruction problem, which attempts either to reconstruct the complete pathways encoded by a given genomic dataset (in a sense, the pathway holes should to be minimized), or to identify the set of pathways that have at least one associated function annotated (i.e., the naïve mapping approach).

### Integer Programming Algorithm

We use integer programming to solve the minimal pathway reconstruction problem. Linear programming (LP) is an algorithm for finding the maximum or minimum of a linear function of variables (objective function) that are subject to linear constraints [24]. Simplex and interior point methods are widely used for solving LP problems. The related problem of integer programming (IP) requires some or all of the variables to take integer (whole number) values. Some of the most powerful algorithms for finding exact solutions of combinatorial optimization problems [25] are based on IP. LP and IP have been applied to many fields in the biological sciences, such as the maximum contact map overlap problem for protein structure comparison [26], optimal protein threading [27], probe design for microarray experiments [28], and the pathway variant problem [8].

Here we transform the minimal pathway reconstruction problem to an integer programming problem: Denote the number of functions (protein families) that are annotated in a dataset as *n*. Let the total number of putative pathways which have at least one component function annotated be *p*. Denote the mapping of protein functions to the pathways as *M*, where *M_ij_* = 1 if function *i* is involved in pathway *j*, otherwise 0 (note one function may map to multiple pathways or subsystems). Denote if a pathway *j* is selected in the final list or not as *P_j_*, with *P_j_* = 1 if selected, *P_j_* = 0 otherwise. The set of pathways with *P_i_* = 1 composes the minimal set of pathways that can explain all the functions that are annotated for a dataset.

The objective function for integer programming is,
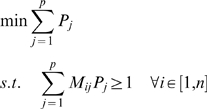
i.e., our goal is to find the minimum number of pathways that can explain all the functions carried by at least one protein from a dataset.

### Protein Function and Function Annotation

We use the KO and FIG protein families defined in the KEGG database and the SEED subsystems, respectively, for this study. Many of the mappings of KO families to KEGG pathways were done manually in the KEGG database. These families are the basic units for pathway reconstruction (or subsystem reconstruction in SEED), in which a pathway (or a subsystem) is composed of a list of functional roles.

### Implementation Details

We use the GLPK package (GNU Linear Programming Kit; http://www.gnu.org/software/glpk/glpk.html) for solving the integer-programming problem; all the other functions are implemented in Python.

The input for MinPath is a list of protein families (*e.g.*, KO and FIG families) annotated in a given dataset of genes (from a genome, or a metagenome), and the output is the list of pathways reconstructed/inferred for the dataset.

Note that in some cases two pathways may share most of their functional roles (for example, the biosynthesis and degradation pathway of the same biological molecule, such as the lysine biosynthesis and degradation pathways). MinPath will keep one of these pathways, because that is sufficient to explain the functional roles identified. We added a post-processing step here to add those pathways that have more than 50% of their functional roles identified back to the pathway pool, even when these functional roles appear in another pathway that is already predicted by MinPath.

### Benchmarking Experiments

We revisited the pathway reconstruction for the 854 genomes in the KEGG database (as of December, 2008) that have at least 20 KEGG pathways annotated for each of these genomes. For these genomes, the function (or protein families) annotations were downloaded from the KEGG database (ftp://ftp.genome.jp/pub/kegg/release/current/).

We also applied MinPath to reanalyze the pathways for nine biome metagenomic datasets [17]. The FIG family annotations for the metagenomic sequences were downloaded from the MG-RAST server (http://metagenomics.theseed.org/). We conducted the KO family annotations of the sequences based on the best blast hits with E-value cutoff of 1e-5, a typical E-value cutoff used for KEGG pathway reconstruction in metagenomes [16].

### Availability and Supplementary Material

MinPath is available as a server and the source codes are available for downloading at MinPath webpage, http://omics.informatics.indiana.edu/MinPath/. Supplementary material is also available at the MinPath website.
